# Radiomic Analysis Based on Abdominal CT-Scan to Predict Strangulation in Adhesive Small Bowel Obstruction: Preliminary Results

**DOI:** 10.3390/jcm14176286

**Published:** 2025-09-05

**Authors:** Francesca Margherita Bunino, Ezio Lanza, Gianluca Sellaro, Riccardo Levi, Davide Zulian, Simone Giudici, Daniele Del Fabbro

**Affiliations:** 1 Department of Biomedical Sciences, Humanitas University, via Rita Levi Montalcini 4, 20072 Pieve Emanuele, MI, Italy; francescabunino@gmail.com (F.M.B.); ezio.lanza@hunimed.eu (E.L.); gianluca.sellaro@humanitas.it (G.S.); riccardo.levi@hunimed.eu (R.L.); 2 Department of Emergency and Trauma Surgery, Fondazione IRCCS Ca’ Granda Ospedale Maggiore Policlinico, via F. Sforza 35, 20122 Milan, MI, Italy; 3 Department of Diagnostic and Interventional Radiology, IRCCS Humanitas Research Hospital, via Manzoni 56, 20089 Rozzano, MI, Italy; 4 Emergency and Trauma Surgery Unit, Department of General Surgery, IRCCS Humanitas Research Hospital, via Manzoni 56, 20089 Rozzano, MI, Italysimone.giudici@humanitas.it (S.G.)

**Keywords:** small bowel obstruction, radiomics, bowel strangulation, emergency surgery

## Abstract

**Introduction:** Small Bowel Obstruction (SBO) accounts for 15% of emergency department (ED) admissions. While conservative management is recommended, surgery becomes necessary when strangulation is suspected. Identifying which patients need surgery remains a challenge, as traditional imaging lacks sufficient sensitivity and specificity. This study aimed to explore radiomic features to identify potential predictors of strangulation. **Methods:** This retrospective study included patients admitted to a tertiary referral hospital ED between 2019 and 2023, diagnosed with Adhesion Small Bowel Obstruction (aSBO) via contrast-enhanced abdominal CT scans. Two patient groups were examined: those who underwent surgery with bowel resection and ischemic changes confirmed histologically (operative management—OM) and those successfully treated with conservative management (CM). All CT scans were reviewed blindly by a general surgeon and an experienced radiologist. Pre-obstructive loop segmentation was performed using 3D Slicer software, with slice-by-slice contouring of intestinal borders on images of suspected strangulated bowel. Radiomic features were extracted, followed by univariate and multivariate regression analysis. **Results:** A total of 55 patients were included: 27 CM and 28 OM. Significant differences emerged in GLCM (Gray Level Co-occurrence Matrix), GLDM (Gray Level Dependence Matrix), GLRLM (Gray Level Run Length Matrix), and GLSZM (Gray Level Size Zone Matrix), particularly involving entropy and uniformity. These metrics reflect subtle variations in gray levels not visible to the naked eye. **Conclusions:** Differences in entropy, uniformity, and energy align with imaging and histopathological findings, supporting the development of radiomic models and future AI-based prediction tools.

## 1. Introduction

Small bowel obstruction (SBO) remains a common and critical cause of acute abdomen, accounting for 15–20% of emergency surgical admissions [1,2,3]. The condition may be mechanical or functional and is often due to adhesions, particularly post-surgical (adhesive SBO or aSBO) [4,5]. Less common causes include hernias, tumors, Crohn’s disease, and gallstone ileus. SBO typically presents with abdominal pain, vomiting, distension, and constipation, and timely diagnosis is essential as strangulation can lead to bowel ischemia, perforation, and mortality rates up to 30% [3,6]. Initial assessment often involves plain abdominal radiography, but its sensitivity is limited. Contrast-enhanced CT is the gold standard, with a sensibility of 94% [7,8,9]. CT features such as mesenteric edema, lack of bowel wall enhancement, pneumatosis intestinalis, the whirl sign, and portal venous gas are key indicators of strangulation or closed-loop obstruction [10,11,12,13,14].

Despite this, one of the persistent challenges in SBO management is distinguishing patients who will improve with conservative treatment from those who require timely surgery. Predictive CT signs and scores (e.g., Angers CT Score) have been developed to help stratify risk and guide intervention [15,16,17,18]. In this field, radiomics offers a promising solution to the limitations of traditional imaging interpretation. This AI-driven approach involves extracting quantitative features—such as shape, texture, and intensity—from standard medical images, transforming them into high-dimensional data that can uncover hidden patterns associated with disease processes [19,20,21].

Originally applied in oncology, radiomics has shown predictive capabilities in areas such as tumor grading, treatment response, and survival outcomes [20,22,23]. In the context of SBO, radiomics may enhance the ability to detect subtle changes in bowel loops and mesentery that suggest ischemia, inflammation, or mechanical tension—features that may not be visible to the naked eye [24,25,26].

This has profound implications not only for improving patient outcomes but also for reducing healthcare costs and avoiding unnecessary surgeries or delayed interventions [13,24,25,26,27,28,29]. The precision offered by radiomics aligns with the growing emphasis on personalized medicine. While many earlier studies have utilized radiomic features to assess disease risk, there are only a limited number focused on intestinal obstruction. Some studies developed basic models to predict strangulated small bowel obstruction based on clinical factors such as average white blood cell (WBC) count, guarding, and CT features indicative of reduced wall enhancement, but these models showed low accuracy [24]. Additionally, a recent study explored the use of radiomics in predicting intestinal strangulation by combining clinical characteristics and radiomic features, aiming to aid emergency and gastrointestinal surgeons in accurately forecasting intestinal necrosis and providing a practical tool for surgical decision making [30]. We opted to validate this model and assess the application of radiomics in our patient population.

## 2. Materials and Methods

### 2.1. Population Definition and Inclusion Process

The conducted study was a retrospective, observational, single-center cohort study involving patients presenting to the Emergency Department (ED) of IRCCS Humanitas Research Hospital, Rozzano, Milan (Italy) between February 2019 and February 2023.

The inclusion criteria were:

- The patient was diagnosed with ASBO.
- The patient was admitted at the IRCCS Humanitas Research Hospital, Rozzano, Milan.
- The patient underwent a diagnostic contrast-enhanced CT scan before being treated.
- The patient was 18 years old or older.
- The patient received a successful conservative management or underwent surgery with bowel resection and histological evidence of bowel ischemia.

The exclusion criteria were:

- The presence of either bowel malignancy, hernia, inflammatory bowel disease (IBD), functional causes of SBO.
- Patients who underwent surgery without bowel resection

**Figure 1:**
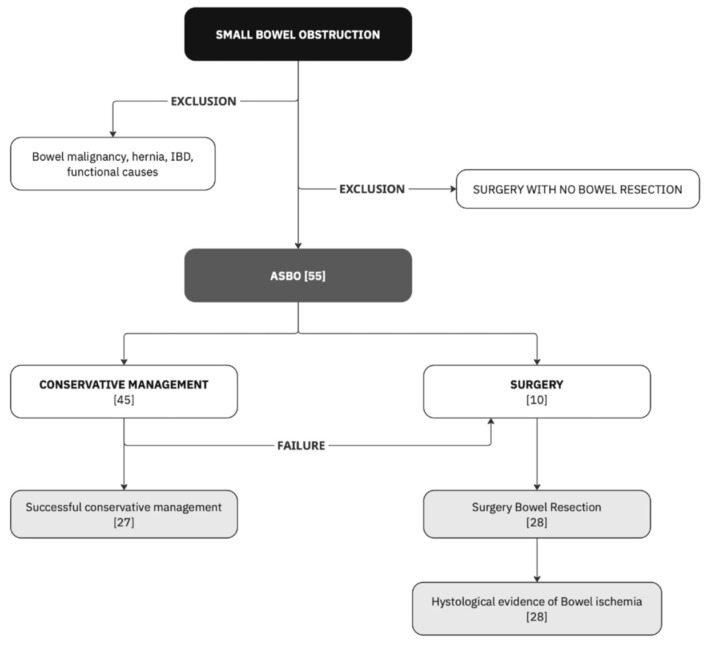
Study inclusion process.

Included patients were stratified, depending on the treatment they received: conservative management group (CM) and operative management group (OM). The choice of treatment was determined based on the surgeon’s professional judgment and the patient’s condition. The first group includes patients who received only conservative treatment, not requiring surgery, while the second group includes patients who underwent a surgical procedure with bowel resection. Within the OM group, all the patients underwent a surgical operation with bowel resection, and the surgical sample was processed and histologically revised with evidence of ischemia. In the CM all the individuals were treated with gastrografin challenge [31] and were successfully discharged both from the ED or from the surgical ward if they required hospitalization. The inclusion process is summarized in Figure 1.

In the diagnostic path, each patient underwent a contrast-enhanced CT scan prior to treatment, according to internally approved guidelines. This was performed using a multidetector CT scanner (Philipps Brilliance 64, Philips Healthcare, Amsterdam, Netherlands), which had the following scanning parameters: tube voltage 120 kV, tube current 130–200 mAs, 240 mAs, collimation 64 × 0.25, pitch 1.4 mm. Both contrast-enhanced and non-contrast images were obtained. An automatic power injection method was used for administering 120–140 mL of iodinated contrast IV (Iomeron 300 mg/mL, Bracco Imaging SpA, Milan, Italy), depending on the patient’s weight. Non-contrast and portal–venous phases after 80 s of contrast administration were acquired. Axial, coronal, and sagittal plane reconstructions of 2.5 mm thickness were then available for examination. Radiation doses followed the Legislative Decree in force at the time of image acquisition.

The outcome was to describe the two groups through their radiomics features outlining differences in the uni- and multivariate analysis. Following previous studies [25] experienced radiologists and surgeons trained in acute care surgery and SBO management assessed several specific CT features relevant to intestinal obstruction. These features included dilated loops, the transition point, wall thickening, increased wall density, ascites, mesenteric fluid, the beak sign, free air, the small intestinal feces sign, the whirlpool sign, and hyperdensity of mesenteric fat.

**Figure 2:**
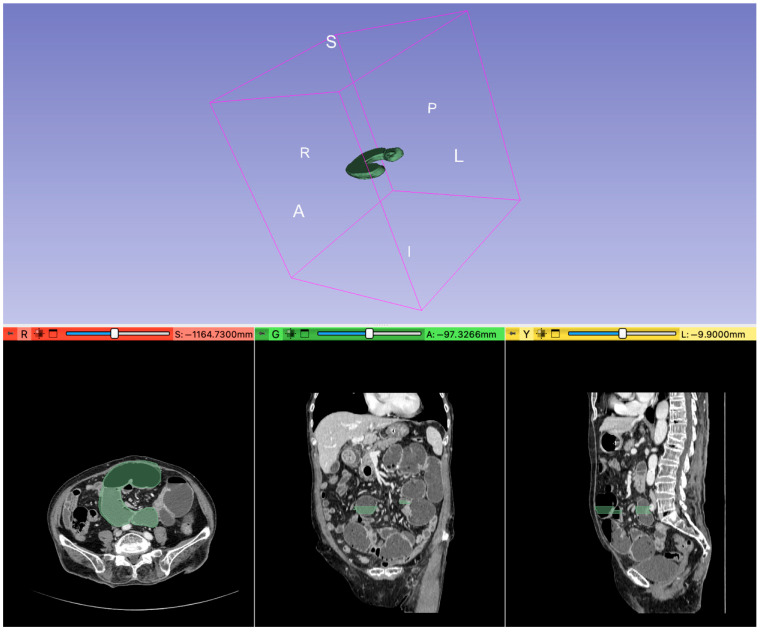
Example of Slicer View for the segmentation drawing.

**Figure 3:**
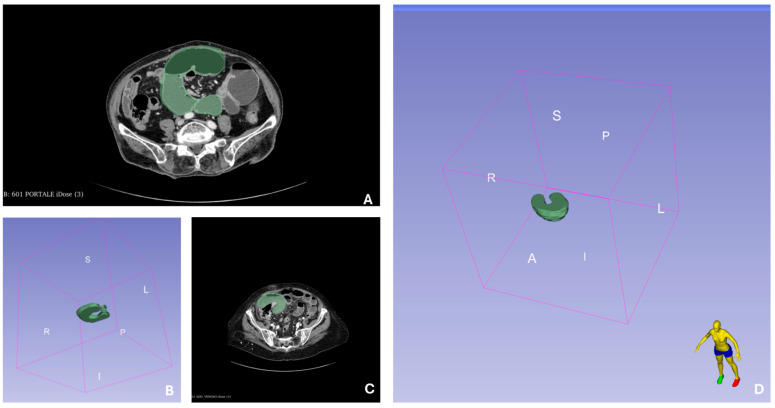
(**A**): Venous phase contrast-enhanced CT scan of a Conservative Management patient with 2D drawn segmentation. (**B**): Three-dimensional reconstruction of the 3-A segmentation with the letters on the cube representing the axes of the figure and the orientation. (**C**): Venous phase contrast-enhanced CT scan of an Operative Management patient with 2D drawn segmentation. (**D**): Three-dimensional reconstruction of the 3-C segmentation with the letters on the cube representing the axes of the figure and the orientation.

Two operators, one surgeon, and one radiologist, unaware of the patient’s clinical details, retrospectively, analyzed the CT images using quantitative region-of-interest (ROI) analysis, a reliable method for diagnosing intestinal ischemia [26]. They performed 3D segmentation of the ROIs within the lesions using 3D Slicer software (version 5.6.2), carefully tracing contours slice-by-slice around the intestinal borders on images of suspected strangulated bowel at the junction of dilated and normal bowel, while avoiding nearby lymph nodes, mesenteries, and other structures and of the dilated bowel proximal to the transition point. An example of the segmentation process is shown in Figure 2. In instances where their drawings differed, the contours were redrawn through discussion. This manual segmentation is illustrated in Figure 3, with A and B representing an example from the OM group (patients with strangulated small bowel obstruction) and Figure 3C,D from the CM group (patients with non-strangulated small bowel obstruction). We then extracted features using the 3D Slicer Radiomics Extension Pack (5.2.2 r31382/fb46bd1). The radiomic characteristics extracted from the suspected strangulated bowel ROIs included first-order characteristics, shape features, and texture features, which describe the morphological and textural attributes of the bowel. In total, we extracted 106 radiomic features, from the 3D Slicer extracted ROIs. For reasons of space, the radiomic features’ description is reported in the Supplementary Material Section in Table S1.

### 2.2. Dataset and Statistical Method

Following previous studies on strangulated bowel obstruction [25], we collected demographic and laboratory variables: sex, age, first episode of SBO, recurrence of SBO, number of previous surgeries, pertinent laboratory parameters (White Blood Cell count [WBC, 103/mL], C-reactive protein [CRP, mg/dL], Urea [mg/dL]), comorbidities and Comorbidities index (Charlson Comorbidity Index(32), both in number and percentage), Intraoperative parameters (Laparoscopic or Open approach, bowel resection, Sigle band or Matted adhesions, stoma fashion) and postoperative variables (complications stratified by Clavien–Dindo scale(33), date of discharge and length of stay, readmission).

The clinical data was methodically extracted from patients’ health records by qualified medical professionals who were not involved in interpreting the CT images of the subjects. The extracted information was then rigorously stored and safeguarded in a database with restricted access.

The sample description was performed using mean and 95% IC for numeric variables (after checking the normality distribution with the Kolgomorov–Smirnov test) and number and proportion for categorical variables. Mann–Whitney and Chi2 tests were used to compare baseline patient characteristics between the two groups, respectively. After data extraction a univariate analysis was conducted to assess the statistical significance for all the variables amongst the groups, considering statistical significance when *p* < 0.05. Secondly, a multivariate logistic regression analysis was conducted considering as confounders the following variables: Charlson, Age, first episode, WBC, CRP and ASA (American Society of Anesthesiology) score.

The standards provided by the 1964 Helsinki Declaration, its later amendments, and the ethical standards of the institutional research committee were respected whenever any procedure involving human subjects was performed. The study was approved by the Institutional Review Board of Humanitas Research Hospital. Informed consent was obtained from each participant prior to the analysis and publication of the reported data.

## 3. Results

### 3.1. Population Characteristics

A total of 56 patients were included in our sample; 27 patients underwent conservative management (CM) and 28 underwent a surgical operation with bowel resection and histological evidence of ischemia (OM). Both groups were equally represented, considering gender and age distribution (respectively, *p* = 0.1851 and 0.9609). No significant differences were reported considering comorbidities (hypertension, diabetes, chronic renal failure, cardiomyopathy, colostomy, and ileostomy rate) and BMI. When looking at the Charlson index percentage, a statistically significant difference was encountered (*p* = 0.0561), while no difference was outlined for the Charlson index number (*p* = 0.0822). No differences were outlined for the frailty score (CFS) and for the ASA score. Considering previous abdominal surgery and in particular the average number of previous laparotomies, no statistical significance was obtained. A total of 16 patients (59%) in the CM group did not have a previous SBO episode (first episode), while in the OM group, there were 25 (89%) (*p* = 0.0325).

No differences were encountered for all the biochemistry blood values (Hb, WBC, Bilirubin, Potassium, Sodium, Creatinine, Urea, CRP, lactate, and Platelets). All the baseline characteristics are available in Table 1.

Radiological variables were then examined; the only statistically significant differences that were outlined between the two groups were related to the small bowel feces sign, which was reported in twenty-five (93%) of CM patients versus fourteen of OMs (50) (*p* = 0.0005) and, to the reduced wall enhancement (just one patient in the CM group versus eighteen patients in the OM) (*p* = 0.002). The radiological variables are presented in Table 2.

All the patients from the CM group were successfully treated with conservative management not requiring abdominal surgery. Of the 28 patients from the OM group, 18 (64%) had a previous attempt at conservative management which failed requiring abdominal surgery. A total of 100% had a bowel resection, of which six of them (21%) were perforated, seventeen (61%) had a single band adhesion, and eight (29%) had matted adhesions. A total of 32% underwent laparoscopic surgery, 18% had a primary laparoscopic approach then converted to open surgery, and finally, 64% underwent open surgery. None of them underwent a stoma fashioning. A total of 13 of the OMs (46%) had a postoperative complication, but just 14% had a Clavien–Dindo complication >3. Mortality was 0 in both groups. A total of 74% of CMs were treated and discharged directly from the ED not requiring hospitalization, and only 4% of CMs had new ED access after being discharged compared to the 11% of the OM group. No significant differences were outlined for the postoperative variable comparison (Table 3).

### 3.2. Radiomics

As previously mentioned in the Materials and Methods, an extended number of radiomic features were extracted from the ROIs and then compared. For reasons of space we decided to report only the variables where a statistically significant difference was reported both at the univariate and multivariate logistic regression. Radiomic comparison results are presented in Table 4. The complete list and analysis are available in the Supplementary Material section (Table S1).

Differences were outlined considering both the Entropy and Uniformity of the two segmentation groups. OMs had a lower level of entropy and more consistent uniformity when compared to CMs (respectively, *p* = 0.0034 and 0.0013 at the univariate and confirmed at the multivariate analysis). When looking at the Correlation and Autocorrelation, which are both expressions of the measure of the magnitude of the fineness and coarseness of the segmentation image, this was higher in the CM group (0.712 versus 0.589, with a *p* = 0.0342 at the univariate and 0.0552 at the multivariate analysis). Energy is a measure of homogeneous patterns in the image. A greater Energy implies that there are more instances of intensity value pairs in the image that neighbor each other at higher frequencies. A higher level of Joint_energy resulted in the OM group when compared to CM (univariate *p* = 0.0026, multivariate *p* = 0.0201). High gray-level emphasis measures the distribution of the higher gray-level values, with a higher value indicating a greater concentration of high gray-level values in the image. This parameter was higher in the CM group with significance both in the uni- and multivariate analyses. All the other features that were evaluated show significant differences between the segmentation volume of the two groups, showing that there are differences in the shape, texture, and composition. Those differences are not visible to the human eye and highlight the limits of traditional radiology even when performed by expert physicians. When translated into mathematic calculations, multiple significant differences were outlined and are reported in the table below. Shape features like elongation or flatness can help distinguish between simple and complex obstructions. In SBO, abnormal mass-like growths or external compression could manifest as irregularities in the shape of bowel loops or adhesions. In our results, the CM group could display greater abnormalities in shape descriptors (such as elongation or sphericity), likely due to the more complex anatomical distortions seen in patients who required surgery. Speaking about texture-based features (GLCM, GLRLM), which quantify tissue heterogeneity, many significant differences were outlined. In the OM group, texture-based radiomic features (e.g., homogeneity, entropy) were significantly different from the CM group, indicating more structural complexity and heterogeneity in the tissues that required surgical intervention. Parameters like skewness and kurtosis, which describe the distribution of pixel intensities, reflecting the underlying structure of the bowel wall and surrounding tissues, were not different in the two examined groups.

## 4. Discussion

Bowel obstruction is still an open field, even if a large number of scientific papers and studies are focused on this topic [32]. As surgeons we have been taught through our learning path that it is crucial to know when not to operate rather than how to operate. This is why we kept on trying to develop more detailed diagnostic scores and to interrogate radiologists for more accurate imaging readings. Additionally, we developed a tailored approach for interpreting contrast-enhanced abdominal CT scans, specifically focusing on identifying indicators such as the whirl sign, transition points, and small bowel fecalization. These key features guide our decision-making process on whether to proceed directly to surgery or attempt conservative management. The existing literature provides substantial evidence regarding when, where, and how to implement conservative management in clinical practice, as well as when it may be deemed unsuccessful. Nevertheless, some aspects still rely on uncertainty [5,25,33,34]. To tackle this issue, we partnered with the radiologists at our institution to investigate the potential for identifying a more precise and effective diagnostic tool. This collaboration aims to enhance our understanding and reduce ambiguity in decision-making processes.

Radiomics has shown promising potential across various areas of medical imaging. Although comprehensive literature specifically addressing radiomics in SBO is limited, its effectiveness in other gastrointestinal disorders, such as Crohn’s disease, implies a promising role [13]. Currently, even if CT has proven to be highly effective for SBO detection, the incorporation of radiomics could further enhance diagnostic accuracy by extracting features from images that may not be easily observed by the human eye, such as texture, shape, and intensity of specific regions [31,35]. These features could aid in identifying specific signs of SBO and its complications, including ischemia and closed-loop obstructions. In clinical settings, the integration of radiomics into SBO diagnosis may help differentiate between cases requiring surgical intervention and those that can be managed conservatively. Radiomic models might provide predictions about the severity of the obstruction or the likelihood of complications like strangulation, potentially surpassing traditional visual assessments. This approach could lead to more tailored treatment strategies and minimize unnecessary surgical procedures and it has already been used in several surgical fields [13,23,36,37].

To better discuss the radiomic results from our analysis and relate them to clinical practice, we will focus on the clinical utility of radiomic features for enhancing diagnosis, prognosis, and personalized treatment [28].

Shape features like elongation or flatness can help distinguish between simple and complex obstructions. In SBO, abnormal mass-like growths or external compression could manifest as irregularities in the shape of bowel loops or adhesions. Detecting these through radiomic analysis could improve the early identification of the obstruction’s cause and guide intervention. In cases of SBO where there is suspicion of mass- or tumor-induced obstruction, these shape-based radiomic metrics could help clinicians decide whether surgical intervention is warranted or if conservative management is possible. No differences were outlined in the two groups. This might be because single and matted adhesion creates a mass effect.

Texture-based features quantify tissue heterogeneity, which is crucial in identifying ischemic bowel segments or areas at risk of necrosis, providing a more quantitative measure compared to the wall thickening detected with the CT scan. In our study, many differences were outlined for shape features, suggesting that the imaging can be strongly predictive for wall necrosis even when not detectable by the naked eye. In the OM group, texture-based radiomic features (e.g., homogeneity, entropy) were significantly different from the CM group, indicating more structural complexity and heterogeneity in the tissues that required surgical intervention. These findings suggest that patients who underwent surgery likely had more severe or advanced disease states, as reflected in the greater heterogeneity of their imaging features. In SBO, for instance, this could indicate the presence of ischemia or strangulation.

Furthermore, the OM group could display greater abnormalities in shape descriptors (such as elongation or sphericity), likely due to the more complex anatomical distortions seen in patients who required surgery.

Shape irregularities are common in patients with obstructive masses, adhesions, or tumors. These features help in understanding the decision to operate, as abnormal shapes often indicate structural complications that cannot be resolved without surgical intervention.

Parameters like skewness and kurtosis, which describe the distribution of pixel intensities, can reflect the underlying structure of the bowel wall and surrounding tissues. In SBO, these metrics could reveal subtle signs of edema, inflammation, or even early ischemia that traditional imaging might not capture with the same precision. In our research, no particular differences were pointed out. This could be considered a limit related to the retrospective nature of the study, to the low number of cases, and finally, to a selection bias.

Lastly, starting from the current univariate and multivariate analyses, a predictive model for SBO severity could be created. In the OM group, multivariate models combining multiple radiomic features (such as intensity, texture, and wavelet features) likely showed higher predictive accuracy in identifying patients needing surgical intervention. Multivariate models incorporating multiple radiomic features (e.g., texture, shape, intensity) could outperform traditional clinical assessments in predicting patient outcomes, such as the need for surgery or the likelihood of postoperative complications. In practice, integrating radiomics into standard clinical workflows could provide a more comprehensive risk stratification for patients with SBO, helping to tailor management strategies based on individual risk profiles. This could prevent unnecessary surgeries in low-risk patients while ensuring high-risk patients receive timely interventions.

Looking at intensity-based features, patients in the OM group likely exhibited significant differences in intensity-based features (e.g., mean intensity, standard deviation) compared to the CM group, reflecting changes in tissue density that might signal necrosis or tissue death. These differences could correspond to early signs of complications such as bowel ischemia in SBO, helping to distinguish cases that would benefit from immediate surgical intervention.

Summarizing, the Conservative Management group might display more homogeneous and less complex radiomic features, suggesting that their conditions were less severe and manageable through conservative means, such as decompression or medication. On the contrary, the operative management group’s radiomic profile likely showed more pronounced structural abnormalities, consistent with the need for surgical correction of anatomical or pathological abnormalities, such as resection of necrotic bowel segments. These differences highlight the potential for using radiomic features to stratify patients based on the severity of their condition, guiding clinical decisions such as whether to pursue surgery or continue with conservative management.

### Limitations

The primary limitation of our study lies in its retrospective design and the limited number of cases analyzed. This restriction arose from the scarcity of patients who underwent bowel resection. Nonetheless, the main objective of this study was to examine the two distinct populations through a radiomic lens to validate a future nomogram and expand the use of this tool to larger populations, ideally on a multicentric scale.

## 5. Conclusions

Radiomics introduces an objective dimension to the subjective nature of image interpretation, particularly beneficial in diagnosing small bowel obstruction (SBO), where timely clinical decisions are crucial. By offering standardized assessments of critical imaging features, radiomics enhances diagnostic accuracy. It provides quantitative insights into variables such as bowel wall thickness, ischemia, and transition points, enabling earlier and more precise diagnoses, which can help mitigate complications and inform treatment strategies. The results from our descriptive statistics about radiomic features highlight potential differences between the examined population which open the field for further studies, potentially on a multicentric stage. Furthermore, the future integration of radiomics with artificial intelligence and machine learning could automate SBO detection, further enhancing diagnostic efficiency. Overall, radiomics serves as a powerful tool for improving SBO diagnosis and management, leading to more data-driven clinical decisions.

## Figures and Tables

**Table 1:** Preoperative variables.

	Conservative Management (CM)	Operative Management (OM)	*p*-Values
	N = 27	N = 28	
**Preoperative Variables**			
Males n (%)	16 (59)	11 (39)	0.19
Age mean (95%IC)	68.750 (41.6–88.4)	68.957 (40.5–89.7)	0.96
1st Episode n (%)	16 (59)	25 (89)	0.03
Colostomy n (%)	3 (11)	0.0	1.00
Ileostomy n (%)	0.0	0.0	1.00
Hypertension n (%)	13 (48)	8 (29)	0.46
Diabetes n(%)	2 (7)	6 (21)	0.17
More than 5 medication intake n(%)	6 (22)	11 (39)	0.47
CRF n(%)	0.0	3 (11)	0.45
Cardiopathy n(%)	4 (15)	8 (29)	0.25
Previous Abdominal surgery n (%)	22 (81)	21 (75)	1.00
Previous laparotomies mean (95%IC)	1.19 (0.80,1.58)	0.64 (0.30,0.98)	0.09
Hb mean (95%IC)	14.16 (8.6–18.38)	14.57 (11.23–17.44)	0.55
PLT mean (95%IC)	297.13 (170.68–628.55)	282.39 (116.90–525.45)	0.92
WBC mean (95%IC)	10.84 (5.36–19.73)	12.26 (6.58–18.61)	0.21
CPR mean (95%IC)	1.87 (0.09–10.12)	6.06 (0.08–25.99)	0.86
Bil Tot mean (95%IC)	1.26 (0.45–3.38)	1.27 (0.31–4.87)	0.53
Urea mean (95%IC)	56.51 (22.56–149.16)	59.65 (10.08–171.79)	0.98
Sodium mean (95%IC)	137.25 (130.88–141.85)	137.0 (131.0–143.35)	0.81
Potassium mean (95%IC)	3.935 (3.03–4.60)	4.13 (3.35–5.19)	0.32
Creatinine mean (95%IC)	0.98 (0.65–1.70)	1.36 (0.57–4.75)	0.44
Lactate mean (95%IC)	2.16 (0.93–3.00)	2.42 (1.33–3.84)	0.68
Charlson index mean (95%IC)	0.67 (0.12–1.22)	2.18 (1.41–2.95)	0.08
Charlson index percentage mean (95%IC)	39.7 (29.61–49.79)	17.18 (4.51–29.85)	0.06
CFS mean (95%IC)	3.20 (2.25–4.15)	2.46 (1.97–2.95)	0.34
ASA median (95%IC)	2 (1.5–2.0)	2 (1.0–3.0)	0.81
BMI mean (95%IC)	20.65 (18.73–23.78)	25.01 (18.75–36.98)	0.09

CRF: Chronic Renal Failure; Hb: Hemoglobin; PLT: Platelet; WBC: White Blood Cell; CRP: C-reactive Protein; Bil: Bilirubin; CFS: Clinical frailty score; ASA: American Society Anesthesiologists; BMI: Body Mass Index.

**Table 2:** Radiological variables.

	Conservative Management (CM)	Operative Management (OM)	*p*-Values
	**N = 27**	**N = 28**	
Abdominal US n (%)	9 (33)	9 (32)	0.61
Complete SBO n (%)	9 (33)	14 (50)	0.41
Small bowel faces sign n (%)	25 (93)	14 (50)	0.00
Pneumatosis n (%)	1 (4)	11 (39)	0.22
Single transition zone n (%)	22 (81)	22 (79)	1.00
Double transition zone n (%)	4 (15)	10 (36)	1.00
Whirls Sign n (%)	5 (18)	6 (21)	1.00
Beak sign n (%)	3 (11)	7 (25)	1.00
Fat Notch sign n (%)	4 (15)	7 (25)	0.65
Reduced wall enhancement n (%)	1 (4)	18 (64)	0.00
Peritoineal fluid n (%)	15 (16)	11 (39)	0.95

**Table 3:** Operative variables. **VLS:** Videolaparoscopy; **LPT:** Laparotomy; **ED:** Emergency Department. **Na**: Not available.

	Conservative Management (CM)	Operative Management (OM)	
	**N = 27**	**N = 28**	
**Conservative management n (%)**	27 (100)	18 (64)	0
**Conservative managmenet failure n (%)**	1 (4)	18 (64)	0.35
**Surgical procedure after failure n (%)**	1 (4)	18 (64)	0.46
**Surgical intervention n (%)**	1 (4)	28 (100)	0
**Bowel resection n (%)**	Na	28 (100)	1
**Bowel perforation n (%)**	Na	6 (21)	1
**Single band sdhesion n (%)**	Na	17 (61)	1
**Mattox adhesions n (%)**	Na	8 (29)	1
**Laparoscopic approach n (%)**	Na	9 (32)	1
**Laparotomy n (%)**	Na	18 (64)	1
**Open Abdomen n (%)**	Na	5 (18)	1
**Conversion from VLS to LPT n (%)**	Na	18.0	1
**Stoma n (%)**	Na	0.0	1
**Complications n (%)**	0.0	13 (46)	0.34
**Clavien dindo** **≥** **3 n (%)**	0.0	4 (14)	1
**Mortality n (%)**	0.0	0.0	0.98
**Discharged from the ED n (%)**	20 (74)	0.0	0
**New ED access within 5 days n (%)**	1 (4)	3 (11)	0.57

**Table 4:** Univariate and multivariate logistic regression results.

Feature	Conservative Management (CM)	Operative Management (OM)	*p*-Values	OR–95%CI	*p*-Value
			**Univariate**	**Multivariate**	
Firstorder_Entropy	3.26 (2.32–4.46)	2.70 (1.94–3.76)	0.01	0.33 (0.13–0.81)	0.02
Firstorder_Minimum	−940.96 (−1024.00–−572.00)	−627.13 (−1024.00–−32.45)	0.02	2.79 (1.10–7.08)	0.03
Firstorder_Uniformity	0.16 (0.07–0.27)	0.22 (0.12–0.34)	0.01	3.41 (1.36–8.54)	0.01
Glcm_Autocorrelation	1409.25 (590.33–1774.64)	851.86 (14.00–1770.97)	0.03	0.45 (0.20–1.00)	0.05
Glcm_Clustershade	−10,938.70 (−36,164.72–739.90)	−3914.69 (−34,569.41–12,015.40)	0.01	2.69 (0.98–7.42)	0.06
Glcm_Correlation	0.71 (0.37–0.93)	0.59 (0.35–0.94)	0.03	0.47 (0.22–1.02)	0.06
Glcm_Difference entropy	2.43 (1.77–3.38)	2.04 (1.38–3.43)	0.01	0.45 (0.20–1.02)	0.06
Glcm_Imc2	0.76 (0.52–0.94)	0.64 (0.42–0.91)	0.01	0.43 (0.19–0.96)	0.04
Glcm_Joint average	36.15 (23.40–41.95)	24.49 (3.55–41.76)	0.04	0.40 (0.17–0.93)	0.03
Glcm_Joint energy	0.05 (0.01–0.10)	0.07 (0.03–0.16)	0.01	3.36 (1.21–9.32)	0.02
**Glcm_Joint entropy**	5.91 (4.27–7.95)	4.94 (3.42–7.25)	0.01	0.35 (0.15–0.86)	0.02
Glcm_Maximum probability	0.12 (0.04–0.24)	0.17 (0.08–0.33)	0.06	2.79 (1.11–6.96)	0.03
Glcm_Sum average	72.29 (46.80–83.90)	48.98 (7.10–83.52)	0.04	0.40 (0.17–0.93)	0.03
Glcm_Sum entropy	4.10 (2.91–5.51)	3.44 (2.54–5.10)	0.01	0.38 (0.16–0.87)	0.02
Gldm_Dependence entropy	6.91 (6.12–7.86)	6.43 (5.87–7.49)	0.01	0.32 (0.13–0.78)	0.01
Gldm_High gray level emphasis	1429.80 (589.43–1782.48)	871.06 (15.57–1765.87)	0.03	0.45 (0.20–1.01)	0.05
Gldm_Small dependence emphasis	0.10 (0.06–0.19)	0.08 (0.04–0.15)	0.03	0.38 (0.15–0.98)	0.04
Gldm_Small dependence high gray level emphasis	124.16 (33.24–207.78)	69.26 (1.21–161.38)	0.01	0.34 (0.14–0.84)	0.02
Glrlm_Gray level non uniformity normalized	0.14 (0.06–0.22)	0.19 (0.09–0.28)	0.01	3.21 (1.29–7.96)	0.01
Glrlm_High gray level run emphasis	1424.63 (590.46–1775.01)	870.73 (16.23–1755.96)	0.02	0.44 (0.20–1.00)	0.05
Glrlm_Long run emphasis	2.65 (1.78–3.63)	3.27 (2.05–6.11)	0.10	2.64 (1.01–6.89)	0.05
Glrlm_Run entropy	4.50 (3.74–5.54)	4.04 (3.35–5.28)	0.01	0.38 (0.16–0.91)	0.03
Glrlm_Short run high gray level emphasis	1148.09 (453.31–1405.24)	698.33 (13.42–1430.10)	0.03	0.44 (0.19–0.99)	0.05
Glszm_Gray level non uniformity normalized	0.06 (0.03–0.13)	0.11 (0.03–0.21)	0.01	2.91 (1.18–7.16)	0.02
Glszm_Low gray level zone emphasis	0.01 (0.00–0.02)	0.03 (0.00–0.20)	0.06	24.36 (0.98–608.45)	0.05
Glszm_Size zone non uniformity	2673.05 (571.60–5480.80)	1657.94 (214.35–5560.23)	0.01	0.46 (0.21–1.00)	0.05
Glszm_Size zone non uniformity normalized	0.403 (0.334–0.504)	0.35 (0.22–0.48)	0.01	0.31 (0.11–0.85)	0.02
Glszm_Small area emphasis	0.658 (0.598–0.737)	0.60 (0.47–0.72)	0.01	0.29 (0.10–0.83)	0.02
Glszm_Zone entropy	6.539 (5.436–7.194)	6.06 (5.12–7.42)	0.01	0.45 (0.20–0.97)	0.04
Glszm_Zone percentage	0.099 (0.046–0.202)	0.07 (0.02–0.16)	0.03	0.41 (0.16–1.02)	0.05

For reasons of space and to facilitate reading, only the statistically significant results were presented in the current table. The full univariate and multivariate results are available in the Supplementary Material section and supplementary table. Statistically significant *p*-values are reported in bold font. **GLCM: Gray Level Co-occurrence Matrix; GLDM: Gray Level Dependence Matrix; GLRLM: Gray Level Run Length Matrix; GLSZM: Gray Level Size Zone; GLSZM: Gray Level Size Zone.**

## Data Availability

The raw data supporting the conclusions of this article are contained within the Supplementary Material. Further inquiries can be directed to the corresponding author.

## Supplementary Materials

The following supporting information can be downloaded at: https://www.mdpi.com/article/10.3390/jcm14176286/s1, Table S1: Complete univariate and multivariate analysis of radiomic features.; Radiomic Features description list.

## Abbreviations

The following abbreviations are used in this manuscript:

SBO	Small Bowel Obstruction
CT	Computed Tomography
WBC	White Blood Cells
ED	Emergency Department
IRCCS	Istituto di Ricovero e Cura a Carattere Scientifico
IBD	Inflammatory Bowel Disease
CM	Conservative management
OM	Operative Management
ROI	Region-Of-Interest
CRP	C-Reactive Protein
ASA	American Society of Anesthesiology
BMI	Body Mass Index
CFS	Clinical Frailty Score
OA	Open Abdomen
VLS	Laparoscopic Surgery
CD	Claviend–Dindo
GLCM	Gray Level Co-occurrence Matrix
GLRLM	Gray Level Run Length Matrix

## References

[1] Maung A.A., Johnson D.C., Piper G.L., Barbosa R.R., Rowell S.E., Bokhari F., Collins J.N., Gordon J.R., Ra J.H., Kerwin A.J. (2012). Evaluation and management of small-bowel obstruction: An Eastern Association for the Surgery of Trauma practice management guideline. J. Trauma Acute Care Surg..

[2] Long B., Robertson J., Koyfman A. (2019). Emergency Medicine Evaluation and Management of Small Bowel Obstruction: Evidence-Based Recommendations. J. Emerg. Med..

[3] Catena F., De Simone B., Coccolini F., Di Saverio S., Sartelli M., Ansaloni L. (2019). Bowel obstruction: A narrative review for all physicians. World J. Emerg. Surg..

[4] Ten Broek R.P.G., Krielen P., Di Saverio S., Coccolini F., Biffl W.L., Ansaloni L., Velmahos G.C., Sartelli M., Fraga G.P., Kelly M.D. (2018). Bologna guidelines for diagnosis and management of adhesive small bowel obstruction (ASBO): 2017 update of the evidence-based guidelines from the world society of emergency surgery ASBO working group. World J. Emerg. Surg..

[5] Catena F., Di Saverio S., Coccolini F., Ansaloni L., De Simone B., Sartelli M., Van Goor H. (2016). Adhesive small bowel adhesions obstruction: Evolutions in diagnosis, management and prevention. World J. Gastrointest. Surg..

[6] Long B., Gottlieb M. (2021). Accuracy of Ultrasonography for the Diagnosis of Small Bowel Obstruction. Am. Fam. Physician.

[7] Kim J.H., Ha H.K., Kim J.K., Eun H.W., Park K.B., Kim B.S., Kim T.K., Kim J.C., Auh Y.H. (2004). Usefulness of Known Computed Tomography and Clinical Criteria for Diagnosing Strangulation in Small-Bowel Obstruction: Analysis of True and False Interpretation Groups in Computed Tomography. World J. Surg..

[8] Zamary K., Spain D.A. (2020). Small Bowel Obstruction: The Sun Also Rises?. J. Gastrointest. Surg..

[9] Nicolaou S., Kai B., Ho S., Su J., Ahamed K. (2005). Imaging of Acute Small-Bowel Obstruction. Am. J. Roentgenol..

[10] Millet I., Taourel P., Ruyer A., Molinari N. (2015). Value of CT findings to predict surgical ischemia in small bowel obstruction: A systematic review and meta-analysis. Eur. Radiol..

[11] Aka A.A., Wright J.P., DeBeche-Adams T. (2021). Small Bowel Obstruction. Clin. Colon Rectal Surg..

[12] Guerrini J., Zugna D., Poretti D., Samà L., Costa G., Mei S., Ceolin M., Biloslavo A., Zago M., Viganò L. (2021). Adhesive small bowel obstruction: Single band or matted adhesions? A predictive model based on computed tomography scan. J. Trauma Acute Care Surg..

[13] Li X., Zhang N., Hu C., Lin Y., Li J., Li Z., Cui E., Shi L., Zhuang X., Li J. (2023). CT-based radiomics signature of visceral adipose tissue for prediction of disease progression in patients with Crohn’s disease: A multicentre cohort study. eClinicalMedicine.

[14] Khurana B. (2003). The Whirl Sign. Radiology.

[15] Zins M., Millet I., Taourel P. (2020). Adhesive Small Bowel Obstruction: Predictive Radiology to Improve Patient Management. Radiology.

[16] Kim J., Lee Y., Yoon J.-H., Lee H.-J., Lim Y.-J., Yi J., Jung W.B. (2021). Non-strangulated adhesive small bowel obstruction: CT findings predicting outcome of conservative treatment. Eur. Radiol..

[17] Kim H.R., Lee Y., Kim J., Baek T.W., Kim H., Son J.H., Park E.J., Kim S.H. (2023). Closed loop obstruction of small bowel: CT signs predicting successful non-surgical treatment. Eur. J. Radiol..

[18] Paisant A., Burgmaier J., Calame P., Loison M., Molière S., Brigand C., Belabbas D., Duchalais E., Regimbeau J., Yzet T. (2023). The Angers CT Score is a Risk Factor for the Failure of the Conservative Management of Adhesive Small Bowel Obstruction: A Prospective Observational Multicentric Study. World J. Surg..

[19] Gillies R.J., Kinahan P.E., Hricak H. (2016). Radiomics: Images Are More than Pictures, They Are Data. Radiology.

[20] Zeynalova A., Kocak B., Durmaz E.S., Comunoglu N., Ozcan K., Ozcan G., Turk O., Tanriover N., Kocer N., Kizilkilic O. (2019). Preoperative evaluation of tumour consistency in pituitary macroadenomas: A machine learning-based histogram analysis on conventional T2-weighted MRI. Neuroradiology.

[21] Savadjiev P., Chong J., Dohan A., Vakalopoulou M., Reinhold C., Paragios N., Gallix B. (2019). Demystification of AI-driven medical image interpretation: Past, present and future. Eur. Radiol..

[22] Mannil M., Von Spiczak J., Muehlematter U.J., Thanabalasingam A., Keller D.I., Manka R., Alkadhi H. (2019). Texture analysis of myocardial infarction in CT: Comparison with visual analysis and impact of iterative reconstruction. Eur. J. Radiol..

[23] Yang L., Yang J., Zhou X., Huang L., Zhao W., Wang T., Zhuang J., Tian J. (2019). Development of a radiomics nomogram based on the 2D and 3D CT features to predict the survival of non-small cell lung cancer patients. Eur. Radiol..

[24] Wang Z., Liu R., Liu S., Sun B., Xie W., Wang D., Lu Y. (2024). A computed tomography-based radiomic model for the prediction of strangulation risk in patients with acute intestinal obstruction. Intell. Med..

[25] Desiato E., Lucia A.M.A., Giudici S., Ammirabile A., Francone M., Lanza E., Del Fabbro D. (2024). Prognostic value of CT findings for conservative treatment failure in adhesive small bowel obstruction. Emerg. Radiol..

[26] Lafata K.J., Wang Y., Konkel B., Yin F.-F., Bashir M.R. (2021). Radiomics: A primer on high-throughput image phenotyping. Abdom. Radiol..

[27] McCague C., Ramlee S., Reinius M., Selby I., Hulse D., Piyatissa P., Bura V., Crispin-Ortuzar M., Sala E., Woitek R. (2023). Introduction to radiomics for a clinical audience. Clin. Radiol..

[28] Bi W.L., Hosny A., Schabath M.B., Giger M.L., Birkbak N.J., Mehrtash A., Allison T., Arnaout O., Abbosh C., Dunn I.F. (2019). Artificial intelligence in cancer imaging: Clinical challenges and applications. CA A Cancer J. Clin..

[29] Jancelewicz T., Vu L.T., Shawo A.E., Yeh B., Gasper W.J., Harris H.W. (2009). Predicting Strangulated Small Bowel Obstruction: An Old Problem Revisited. J. Gastrointest. Surg..

[30] D’Agostino R., Ali N.S., Leshchinskiy S., Cherukuri A.R., Tam J.K. (2018). Small bowel obstruction and the gastrografin challenge. Abdom. Radiol..

[31] Charlson M.E., Pompei P., Ales K.L., MacKenzie C.R. (1987). A new method of classifying prognostic comorbidity in longitudinal studies: Development and validation. J. Chronic Dis..

[32] Clavien P.A., Barkun J., de Oliveira M.L., Vauthey J.N., Dindo D., Schulick R.D., de Santibañes E., Pekolj J., Slankamenac K., Bassi C. (2009). The Clavien-Dindo Classification of Surgical Complications: Five-Year Experience. Ann. Surg..

[33] Rami Reddy S.R., Cappell M.S. (2017). A Systematic Review of the Clinical Presentation, Diagnosis, and Treatment of Small Bowel Obstruction. Curr. Gastroenterol. Rep..

[34] Andersen P., Jensen K.K., Erichsen R., Frøslev T., Krarup P.-M., Madsen M.R., Laurberg S., Iversen L.H. (2017). Nationwide population-based cohort study to assess risk of surgery for adhesive small bowel obstruction following open or laparoscopic rectal cancer resection: Surgery for adhesive small bowel obstruction after rectal cancer resection. BJS Open.

[35] Cohen R.B., Olafson S.N., Krupp J., Parsikia A., Kaplan M.J., Moran B., Leung P.S. (2021). Timing of Gastrografin administration in the management of adhesive small bowel obstruction (ASBO): Does it matter?. Surgery.

[36] Wei Y., Liao T., Shangguan X., Ouyang M., Chen Z., Zheng E., Lin B., Chen X. (2023). A multi-analysis of nomogram model for the identification of banded adhesions and matted adhesions in adhesive small bowel obstruction. Eur. J. Trauma Emerg. Surg..

[37] Zhou X.-Y., Guo Y., Shen M., Yang G.-Z. (2020). Application of artificial intelligence in surgery. Front. Med..

