# Supplementary material for: Radiomic Analysis Based on Abdominal CT-Scan to Predict Strangulation in Adhesive Small Bowel Obstruction: Preliminary Results
Source: J Clin Med. 2025 Sep 5;14(17):6286. doi: 10.3390/jcm14176286 (PMC12429312; doi:10.3390/jcm14176286)
Supplement: Supplementary file 1 [file jcm-14-06286-s001.zip › jcm-3816953-supplementary.pdf]

Radiomic Feature	CM	OM	p-values	OR 95%CI		p-value
			Univariate	Multivariate		
**shape_Elongation**	0,589 (0,346 - 0,830)	0,55 (0,29 - 0,81)	0,46	0,62	(0,29-1,37)	0,24
**shape_Flatness**	0,394 (0,220 - 0,599)	0,37 (0,15 - 0,60)	0,59	0,77	(0,35-1,73)	0,53
**shape_LeastAxisLength**	37,37 (24,29 - 51,04)	36,54 (23,76 - 56,80)	0,73	0,69	(0,32-1,47)	0,34
**shape_MajorAxisLength**	100,82 (65,47 - 155,21)	111,79 (58,86 - 233,21)	0,62	1,32	(0,63-2,74)	0,46
**shape_Maximum2DDiameterColumn**	90,70 (50,06 - 154,65)	103,75 (57,32 - 174,80)	0,17	1,19	(0,53-2,65)	0,67
**shape_Maximum2DDiameterRow**	85,87 (59,62 - 125,82)	83,30 (57,60 - 148,34)	0,46	0,72	(0,31-1,70)	0,46
**shape_Maximum2DDiameterSlice**	105,34 (69,89 - 138,71)	98,61 (49,88 - 166,05)	0,22	0,63	(0,28-1,39)	0,25
**shape_Maximum3DDiameter**	114,08 (76,52 - 154,95)	122,35 (71,28 - 194,78)	0,35	1,02	(0,48-2,16)	0,96
**shape_MeshVolume**	110858,34 (52864,08 - 246322,73)	104552,55 (34233,81 - 268467,27)	0,30	0,69	(0,31-1,56)	0,37
**shape_MinorAxisLength**	56,52 (39,62 - 82,11)	56,96 (35,29 - 100,04)	0,60	0,66	(0,29-1,50)	0,32
**shape_Sphericity**	0,538 (0,469 - 0,622)	0,48 (0,31 - 0,65)	0,04	0,59	(0,26-1,34)	0,21
**shape_SurfaceArea**	20613,33 (12130,84 - 38732,29)	22524,16 (9813,43 - 47393,12)	0,84	0,89	(0,39-2,04)	0,78
**shape_SurfaceVolumeRatio**	0,196 (0,149 - 0,242)	0,24 (0,16 - 0,44)	0,05	2,62	(0,92-7,42)	0,07
**shape_VoxelVolume**	111027,96 (53016,75 - 246530,19)	104833,37 (34373,81 - 268881,56)	0,30	0,69	(0,31-1,56)	0,38
**firstorder_10Percentile**	-268,29 (-982,55 - -10,45)	-140,01 (-987,75 - 12,30)	0,01	1,83	(0,83-4,03)	0,13
**firstorder_90Percentile**	73,58 (48,45 - 94,55)	64,30 (11,95 - 93,80)	0,15	0,46	(0,16-1,31)	0,15
**firstorder_Energy**	3758645746,71 (50174129,00 - 15241991366,13)	3200100239,57 (19753143,50 - 26591727259,35)	0,03	0,90	(0,45-1,78)	0,76
**firstorder_Entropy**	3,26 (2,32 - 4,46)	2,70 (1,94 - 3,76)	0,00	0,33	(0,13-0,81)	0,02
**firstorder_InterquartileRange**	123,52 (30,00 - 741,65)	61,57 (25,55 - 266,45)	0,00	0,36	(0,10-1,25)	0,11
**firstorder_Kurtosis**	23,28 (1,85 - 70,45)	23,38 (3,15 - 82,32)	0,35	0,95	(0,41-2,20)	0,91

**firstorder_Maximum**	208,04 (146,00 - 415,13)	256,35 (132,30 - 781,00)	0,38	1,37 2,87)	(0,65-	0,41
**firstorder_MeanAbsoluteDeviation**	117,93 (22,60 - 396,27)	69,79 (17,38 - 305,30)	0,02	0,47 1,09)	(0,21-	0,08
**firstorder_Mean**	-50,24 (-317,45 - 25,77)	-28,97 (-412,48 - 44,51)	0,01	1,22 2,45)	(0,60-	0,59
**firstorder_Median**	2,54 (-155,58 - 29,70)	-18,96 (-432,15 - 46,35)	0,11	0,80 1,58)	(0,40-	0,52
**firstorder_Minimum**	-940,96 (-1024,00 - -572,00)	-627,13 (-1024,00 - -32,45)	0,02	2,79 7,08)	(1,10-	0,03
**firstorder_Range**	1149,00 (718,00 - 1367,25)	883,48 (236,40 - 1805,00)	0,05	0,54 1,19)	(0,24-	0,13
**firstorder_RobustMeanAbsoluteDeviation**	65,25 (13,42 - 342,07)	36,46 (11,11 - 207,82)	0,02	0,47 1,19)	(0,19-	0,11
**firstorder_RootMeanSquared**	192,31 (37,14 - 546,43)	135,50 (31,95 - 594,30)	0,04	0,65 1,38)	(0,30-	0,26
**firstorder_Skewness**	-3,15 (-6,92 - -0,47)	-2,19 (-6,68 - 1,12)	0,15	1,65 3,56)	(0,77-	0,20
**firstorder_TotalEnergy**	6210890756,29 (95608163,27 - 30238040043,93)	5305930087,83 (49671205,43 - 45657023755,77)	0,03	0,86 1,73)	(0,43-	0,68
**firstorder_Uniformity**	0,162 (0,067 - 0,270)	0,22 (0,12 - 0,34)	0,00	3,41 8,54)	(1,36-	0,01
**firstorder_Variance**	47017,15 (1054,42 - 192331,24)	23889,38 (564,45 - 142989,02)	0,02	0,48 1,10)	(0,21-	0,08
**glcm_Autocorrelation**	1409,25 (590,33 - 1774,64)	851,86 (14,00 - 1770,97)	0,03	0,45 1,00)	(0,20-	0,05
**glcm _ClusterProminence**	841195,53 (167,02 - 2574318,57)	426542,99 (27,53 - 2388471,72)	0,01	0,48 1,12)	(0,21-	0,09
**glcm_ClusterShade**	-10938,70 (-36164,72 - 739,90)	-3914,69 (-34569,41 - 12015,40)	0,01	2,69 7,42)	(0,98-	0,06
**glcm_ClusterTendency**	291,46 (4,20 - 1186,70)	140,45 (2,38 - 820,42)	0,02	0,44 1,05)	(0,19-	0,06
**glcm_Contrast**	21,10 (1,89 - 66,02)	15,23 (0,85 - 97,92)	0,01	0,79 1,62)	(0,39-	0,52
**glcm_Correlation**	0,712 (0,373 - 0,926)	0,59 (0,35 - 0,94)	0,03	0,47 1,02)	(0,22-	0,06
**glcm_DifferenceAverage**	2,06 (0,932 - 4,35)	1,60 (0,61 - 5,46)	0,01	0,68 1,45)	(0,32-	0,32
**glcm_DifferenceEntropy**	2,43 (1,77 - 3,38)	2,04 (1,38 - 3,43)	0,01	0,45 1,02)	(0,20-	0,06
**glcm_DifferenceVariance**	15,48 (1,01 - 45,46)	9,63 (0,47 - 55,15)	0,01	0,64 1,35)	(0,30-	0,24
**glcm_gray level co-occourence matrix_Id**	0,565 (0,445 - 0,645)	0,61 (0,47 - 0,73)	0,03	2,19 5,16)	(0,93-	0,07

**glcm_Idm**	0,525 (0,384 - 0,617)	0,58 (0,42 - 0,72)	0,02	2,22	(0,94- 5,23)	0,07
**glcm_Idmn**	0,993 (0,977 - 0,999)	0,99 (0,97 - 0,99)	0,57	0,99	(0,52- 1,89)	0,98
**glcm_Idn**	0,963 (0,931 - 0,977)	0,96 (0,91 - 0,98)	0,79	0,80	(0,41- 1,56)	0,51
**glcm_Imc1**	-0,163 (-0,286 - -0,079)	-0,13 (-0,26 - -0,05)	0,09	1,78	(0,83- 3,81)	0,14
**glcm_Imc2**	0,755 (0,516 - 0,942)	0,64 (0,42 - 0,91)	0,01	0,43	(0,19- 0,96)	0,04
**glcm_InverseVariance**	0,428 (0,304 - 0,490)	0,45 (0,28 - 0,50)	0,06	1,32	(0,65- 2,69)	0,45
**glcm_JointAverage**	36,15 (23,40 - 41,95)	24,49 (3,55 - 41,76)	0,04	0,40	(0,17- 0,93)	0,03
**glcm_JointEnergy**	0,046 (0,013 - 0,099)	0,07 (0,03 - 0,16)	0,00	3,36	(1,21- 9,32)	0,02
**glcm_JointEntropy**	5,91 (4,27 - 7,95)	4,94 (3,42 - 7,25)	0,00	0,35	(0,15- 0,86)	0,02
**glcm_MCC**	0,759 (0,491 - 0,940)	0,67 (0,40 - 0,96)	0,07	0,54	(0,26- 1,15)	0,11
**glcm_MaximumProbability**	0,123 (0,037 - 0,243)	0,17 (0,08 - 0,33)	0,06	2,79	(1,11- 6,96)	0,03
**glcm_SumAverage**	72,29 (46,80 - 83,90)	48,98 (7,10 - 83,52)	0,04	0,40	(0,17- 0,93)	0,03
**glcm_SumEntropy**	4,10 (2,91 - 5,51)	3,44 (2,54 - 5,10)	0,01	0,38	(0,16- 0,87)	0,02
**glcm_SumSquares**	78,14 (1,52 - 312,01)	38,92 (0,81 - 216,22)	0,02	0,46	(0,20- 1,06)	0,07
**gldm_DependenceEntropy**	6,91 (6,12 - 7,86)	6,43 (5,87 - 7,49)	0,00	0,32	(0,13- 0,78)	0,01
**gldm_DependenceNonUniformity**	4563,66 (1968,85 - 10135,52)	4245,18 (998,26 - 11650,39)	0,28	0,72	(0,32- 1,64)	0,44
**gldm_DependenceNonUniformityNormalized**	0,067 (0,052 - 0,096)	0,07 (0,04 - 0,10)	0,71	0,95	(0,49- 1,86)	0,89
**gldm_DependenceVariance**	22,42 (11,06 - 39,57)	22,56 (10,51 - 44,28)	0,82	1,08	(0,56- 2,07)	0,82
**gldm_GrayLevelNonUniformity**	11303,21 (2774,98 - 26368,73)	13766,55 (4389,26 - 43007,35)	0,48	1,18	(0,42- 3,33)	0,75
**gldm_GrayLevelVariance**	75,29 (1,77 - 307,66)	38,26 (0,99 - 228,57)	0,02	0,48	(0,21- 1,10)	0,08
**gldm_HighGrayLevelEmphasis**	1429,80 (589,43 - 1782,48)	871,06 (15,57 - 1765,87)	0,03	0,45	(0,20- 1,01)	0,05
**gldm_LargeDependenceEmphasis**	86,10 (40,76 - 124,62)	99,26 (52,63 - 189,96)	0,31	1,57	(0,77- 3,22)	0,22
**gldm_LargeDependenceHighGrayLevelEmphasis**	119286,24 (56323,24 - 200815,68)	73474,36 (1127,19 - 177979,75)	0,01	0,53	(0,24- 1,15)	0,11

**gldm_LargeDependenceLowGrayLevelEmphasis**	1,73 (0,039 - 14,22)	4,61 (0,04 - 39,88)	0,22	1,39 2,89)	(0,67-	0,38
**gldm_LowGrayLevelEmphasis**	0,011 (0,001 - 0,068)	0,03 (0,00 - 0,26)	0,13	1,67 4,36)	(0,64-	0,30
**gldm_SmallDependenceEmphasis**	0,104 (0,058 - 0,191)	0,08 (0,04 - 0,15)	0,03	0,38 0,98)	(0,15-	0,04
**gldm_SmallDependenceHighGrayLevelEmphasis**	124,16 (33,24 - 207,78)	69,26 (1,21 - 161,38)	0,01	0,34 0,84)	(0,14-	0,02
**gldm_SmallDependenceLowGrayLevelEmphasis**	0,001 (0,001 - 0,002)	0,00 (0,00 - 0,01)	0,40	2,48 12,92)	(0,48-	0,28
**glrlm_GrayLevelNonUniformity**	7129,90 (1744,79 - 16527,58)	8379,16 (2855,30 - 25123,53)	0,69	1,05 2,73)	(0,40-	0,92
**glrlm_GrayLevelNonUniformityNormalized**	0,140 (0,056 - 0,217)	0,19 (0,09 - 0,28)	0,00	3,21 7,96)	(1,29-	0,01
**glrlm_GrayLevelVariance**	73,29 (2,18 - 269,66)	40,90 (1,22 - 230,17)	0,02	0,53 1,17)	(0,24-	0,12
**glrlm_HighGrayLevelRunEmphasis**	1424,63 (590,46 - 1775,01)	870,73 (16,23 - 1755,96)	0,02	0,44 1,00)	(0,20-	0,05
**glrlm_LongRunEmphasis**	2,65 (1,78 - 3,63)	3,27 (2,05 - 6,11)	0,10	2,64 6,89)	(1,01-	0,05
**glrlm_LongRunHighGrayLevelEmphasis**	3694,42 (1970,56 - 5751,28)	2640,63 (47,43 - 7654,51)	0,04	0,75 1,54)	(0,36-	0,43
**glrlm_LongRunLowGrayLevelEmphasis**	0,043 (0,002 - 0,333)	0,14 (0,00 - 1,13)	0,11	1,56 3,55)	(0,68-	0,29
**glrlm_LowGrayLevelRunEmphasis**	0,009 (0,001 - 0,048)	0,03 (0,00 - 0,23)	0,13	2,09 7,86)	(0,56-	0,27
**glrlm_RunEntropy**	4,50 (3,74 - 5,54)	4,04 (3,35 - 5,28)	0,00	0,38 0,91)	(0,16-	0,03
**glrlm_RunLengthNonUniformity**	31337,48 (13501,72 - 68890,53)	27202,61 (6435,91 - 72695,75)	0,16	0,64 1,47)	(0,28-	0,29
**glrlm_RunLengthNonUniformityNormalized**	0,615 (0,549 - 0,728)	0,59 (0,46 - 0,71)	0,14	0,66 1,38)	(0,31-	0,27
**glrlm_RunPercentage**	0,735 (0,672 - 0,830)	0,71 (0,57 - 0,79)	0,11	0,58 1,24)	(0,27-	0,16
**glrlm_RunVariance**	0,737 (0,310 - 1,404)	1,09 (0,41 - 3,06)	0,14	2,55 6,94)	(0,94-	0,07
**glrlm_ShortRunEmphasis**	0,809 (0,768 - 0,876)	0,79 (0,70 - 0,86)	0,07	0,57 1,26)	(0,26-	0,16
**glrlm_ShortRunHighGrayLevelEmphasis**	1148,09 (453,31 - 1405,24)	698,33 (13,42 - 1430,10)	0,03	0,44 0,99)	(0,19-	0,05
**glrlm_ShortRunLowGrayLevelEmphasis**	0,007 (0,001 - 0,031)	0,02 (0,00 - 0,17)	0,12	2,51 12,12)	(0,52-	0,25
**glszm_GrayLevelNonUniformity**	333,45 (165,03 - 708,69)	364,10 (133,17 - 975,19)	0,91	0,92 2,11)	(0,40-	0,84

**glszm_GrayLevelNonUniformityNormalized**	0,063 (0,026 - 0,128)	0,11 (0,03 - 0,21)	0,00	2,91	(1,18- 7,16)	0,02
**glszm_GrayLevelVariance**	94,44 (7,21 - 181,89)	57,78 (3,03 - 184,69)	0,02	0,52	(0,24- 1,13)	0,10
**glszm_HighGrayLevelZoneEmphasis**	1158,73 (558,87 - 1588,92)	761,74 (20,00 - 1641,73)	0,06	0,52	(0,23- 1,15)	0,11
**glszm_LargeAreaEmphasis**	135236,06 (7839,46 - 403779,02)	260529,05 (22274,50 - 1391398,71)	0,25	1,84	(0,38- 8,89)	0,45
**glszm_LargeAreaHighGrayLevelEmphasis**	201494953,93 (4943361,86 - 713802403,30)	159901477,51 (257819,30 - 762312835,16)	0,19	0,67	(0,28- 1,59)	0,37
**glszm_LargeAreaLowGrayLevelEmphasis**	277,13 (10,89 - 1480,01)	4056,34 (33,10 - 27703,27)	0,04	11,96	(0,92- 156,22)	0,06
**glszm_LowGrayLevelZoneEmphasis**	0,006 (0,001 - 0,016)	0,03 (0,00 - 0,20)	0,06	24,36	(0,98- 608,45)	0,05
**glszm_SizeZoneNonUniformity**	2673,05 (571,60 - 5480,80)	1657,94 (214,35 - 5560,23)	0,01	0,46	(0,21- 1,00)	0,05
**glszm_SizeZoneNonUniformityNormalized**	0,403 (0,334 - 0,504)	0,35 (0,22 - 0,48)	0,00	0,31	(0,11- 0,85)	0,02
**glszm_SmallAreaEmphasis**	0,658 (0,598 - 0,737)	0,60 (0,47 - 0,72)	0,00	0,29	(0,10- 0,83)	0,02
**glszm_SmallAreaHighGrayLevelEmphasis**	729,30 (338,84 - 1005,42)	458,37 (10,73 - 1005,98)	0,04	0,48	(0,21- 1,09)	0,08
**glszm_SmallAreaLowGrayLevelEmphasis**	0,004 (0,001 - 0,010)	0,02 (0,00 - 0,12)	0,11	19,25	(0,81- 458,42)	0,07
**glszm_ZoneEntropy**	6,54 (5,44 - 7,19)	6,06 (5,12 - 7,42)	0,01	0,45	(0,20- 0,97)	0,04
**glszm_ZonePercentage**	0,099 (0,046 - 0,202)	0,07 (0,02 - 0,16)	0,03	0,41	(0,16- 1,02)	0,05
**glszm_ZoneVariance**	135048,03 (7814,83 - 403445,29)	260092,65 (22145,27 - 1389798,62)	0,25	1,83	(0,38- 8,87)	0,45
**ngtdm_Busyness**	3,08 (1,50 - 5,46)	20,88 (1,12 - 95,93)	0,03	9,54	(1,24- 73,44)	0,03
**ngtdm_Coarseness**	0,001 (0,001 - 0,001)	0,00 (0,00 - 0,00)	0,77	1,92	(0,78- 4,68)	0,15
**ngtdm_Complexity**	1745,14 (198,67 - 3424,90)	1107,92 (25,59 - 3742,96)	0,01	0,54	(0,25- 1,16)	0,12
**ngtdm_Contrast**	0,114 (0,003 - 0,520)	0,05 (0,00 - 0,28)	0,15	0,41	(0,15- 1,16)	0,09
**ngtdm_Strength**	0,506 (0,170 - 0,878)	0,48 (0,01 - 1,79)	0,17	1,10	(0,54- 2,24)	0,80

Table S1. Complete univariate and multivariate analysis of radiomic features.

Supplementary Materials

1. *Radiomic Features description list*

The following list reports all the radiomic features that were extracted from the 3D slicer ROIs [39].

- Shape_Elongation: Elongation shows the relationship between the two largest principal components in the ROI shape.
- Shape_Flatness: Flatness shows the relationship between the largest and smallest principal components in the ROI shape
- Shape_LeastAxisLength: λmajor and λleast are the lengths of the largest and smallest principal component axes.
- Shape_MajorAxisLength: λmajor and λleast are the lengths of the largest and smallest principal component axes.
- Shape_Maximum2DDiameterColumn: Maximum diameter is defined as the largest pairwise Euclidean distance between tumor surface mesh vertices.
- Shape_Maximum2DDiameterRow
- Shape_Maximum2DDiameterSlice
- Shape_Maximum3DDiameter
- Shape_MeshVolume: The volume of the ROI V is calculated from the triangle mesh of the ROI
- Shape_MinorAxisLength
- Shape_Sphericity: Sphericity is a measure of the roundness of the Shape of the tumor region relative to a sphere. It is a dimensionless measure, independent of scale and orientation. The value range is $0<sphericity\leq 1$, where a value of 1 indicates a perfect sphere (a sphere has the smallest possible surface area for a given volume, compared to other solids).
- Shape_SurfaceArea
- Shape_SurfaceVolumeRatio
- Shape_VoxelVolume: The volume of the ROI $Vvoxel$ is approximated by multiplying the number of voxels in the ROI by the volume of a single voxel Vk. This is a less precise approximation of the volume and is not used in subsequent features. This feature does not make use of the mesh and is not used in calculation of other shape features.
- Firstorder_10Percentile
- Firstorder_90Percentile
- Firstorder_Energy: Energy is a measure of the magnitude of voxel values in an image. A larger value implies a greater sum of the squares of these values.
- Firstorder_Entropy: Entropy specifies the uncertainty/randomness in the image values. It measures the average amount of information required to encode the image values.
- Firstorder_InterquartileRange
- Firstorder_Kurtosis: Kurtosis is a measure of the 'peakedness' of the distribution of values in the image ROI. A higher kurtosis implies that the mass of the distribution is concentrated towards the tail(s) rather than towards the mean. A lower kurtosis implies the reverse: that the mass of the distribution is concentrated towards a spike near the Mean value.
- Firstorder_Maximum
- Firstorder_MeanAbsoluteDeviation
- Firstorder_Mean
- Firstorder_Median
- Firstorder_Minimum
- Firstorder_Range
- Firstorder_RobustMeanAbsoluteDeviation
- Firstorder_RootMeanSquared

- Firstorder_Skewness: Skewness measures the asymmetry of the distribution of values about the Mean value. Depending on where the tail is elongated and the mass of the distribution is concentrated, this value can be positive or negative.
- Firstorder_Total Energy
- Firstorder_Uniformity: Uniformity is a measure of the sum of the squares of each intensity value. This is a measure of the homogeneity of the image array, where a greater uniformity implies a greater homogeneity or a smaller range of discrete intensity values.
- Firstorder_Variance: Variance is the the mean of the squared distances of each intensity value from the Mean value. This is a measure of the spread of the distribution about the mean. By definition, variance=$\sigma 2$
- GLCM: Grey Level Co-Occourence Matrix is a matrix that is defined over an image to be the distribution of co-occurring pixel values (grayscale values, or colors) at a given offset. It is used as an approach to texture analysis with various applications especially in medical image analysis.
- GLCM_Autocorrelation: Autocorrelation is a measure of the magnitude of the fineness and coarseness of texture.
- ClusterProminence: Cluster Prominence is a measure of the skewness and asymmetry of the GLCM. A higher value implies more asymmetry about the mean while a lower value indicates a peak near the mean value and less variation about the mean.
- GLCM_ClusterShade: Cluster Shade is a measure of the skewness and uniformity of the GLCM. A higher cluster shade implies greater asymmetry about the mean.
- GLCM_ClusterTendency: Cluster Tendency is a measure of groupings of voxels with similar gray-level values.
- GLCM_Contrast: Contrast is a measure of the local intensity variation, favoring values away from the diagonal ($i=j$). A larger value correlates with a greater disparity in intensity values among neighboring voxels.
- GLCM_Correlation: Correlation is a value between 0 (uncorrelated) and 1 (perfectly correlated) showing the linear dependency of gray level values to their respective voxels in the GLCM.
- GLCM_DifferenceAverage: Difference Average measures the relationship between occurrences of pairs with similar intensity values and occurrences of pairs with differing intensity values.
- GLCM_DifferenceEntropy: Difference Entropy is a measure of the randomness/variability in neighborhood intensity value differences.
- GLCM_DifferenceVariance: Difference Variance is a measure of heterogeneity that places higher weights on differing intensity level pairs that deviate more from the mean.
- GLCM _Id
- GLCM_Idm
- GLCM_Idmn
- GLCM_Idn
- GLCM_Imc1
- GLCM_Imc2
- GLCM_InverseVariance
- GLCM_JointAverage Returns the mean gray level intensity of the *i* distribution.
- GLCM_JointEnergy Energy is a measure of homogeneous patterns in the image. A greater Energy implies that there are more instances of intensity value pairs in the image that neighbor each other at higher frequencies.
- GLCM_JointEntropy Joint entropy is a measure of the randomness/variability in neighborhood intensity values.
- GLCM_MCC
- GLCM_MaximumProbability

- GLCM_SumAverage Sum Average measures the relationship between occurrences of pairs with lower intensity values and occurrences of pairs with higher intensity values.
- GLCM_SumEntropy Sum Entropy is a sum of neighborhood intensity value differences.
- GLCM_SumSquares Sum of Squares or Variance is a measure in the distribution of neigboring intensity level pairs about the mean intensity level in the GLCM.
- GLDM: Gray Level Dependence Matrix quantifies gray level dependencies in an image. A gray level dependency is defined as the number of connected voxels within distance δ that are dependent on the center voxel. A neighbouring voxel with gray level j is considered dependent on center voxel with gray level i if $|i-j|\le\alpha$. In a gray level dependence matrix $\mathbf{P}(i,j)$ the (i,j)th element describes the number of times a voxel with gray level i with j dependent voxels in its neighbourhood appears in image.
- GLDM_DependenceEntropy
- GLDM_DependenceNonUniformity: Measures the similarity of dependence throughout the image, with a lower value indicating more homogeneity among dependencies in the image.
- GLDM_DependenceNonUniformityNormalized: Measures the similarity of dependence throughout the image, with a lower value indicating more homogeneity among dependencies in the image. This is the normalized version of the DLN formula.
- GLDM_DependenceVariance: Measures the variance in dependence size in the image.
- GLDM_GrayLevelNonUniformity: GLN measures the variability of gray-level intensity values in the image, with a lower value indicating more homogeneity in intensity values.
- GLDM_GrayLevelVariance: Measures the variance in grey level in the image.
- GLDM_HighGrayLevelEmphasis: Measures the distribution of the higher gray-level values, with a higher value indicating a greater concentration of high gray-level values in the image.
- GLDM_LargeDependenceEmphasis: A measure of the distribution of large dependencies, with a greater value indicative of larger dependence and more homogeneous textures.
- GLDM_LargeDependenceHighGrayLevelEmphasis: Measures the joint distribution of large dependence with higher gray-level values.
- GLDM_LargeDependenceLowGrayLevelEmphasis: Measures the joint distribution of large dependence with lower gray-level values.
- GLDM_LowGrayLevelEmphasis: Measures the distribution of low gray-level values, with a higher value indicating a greater concentration of low gray-level values in the image.
- GLDM_SmallDependenceEmphasis: A measure of the distribution of small dependencies, with a greater value indicative of smaller dependence and less homogeneous textures.
- GLDM_SmallDependenceHighGrayLevelEmphasis: Measures the joint distribution of small dependence with higher gray-level values.
- GLDM_SmallDependenceLowGrayLevelEmphasis: Measures the joint distribution of small dependence with lower gray-level values.
- Gray Level Run Length Matrix (GLRLM): quantifies gray level runs, which are defined as the length in number of pixels, of consecutive pixels that have the same gray level value.
- GLRLM_GrayLevelNonUniformity: Measures the similarity of gray-level intensity values in the image, where a lower GLN value correlates with a greater similarity in intensity values.
- GLRLM_GrayLevelNonUniformityNormalized: GLNN measures the variability of gray-level intensity values in the image, with a lower value indicating a greater similarity in intensity values. This is the normalized version of the GLN formula.
- GLRLM_GrayLevelVariance
- GLRLM_HighGrayLevelRunEmphasis
- GLRLM_LongRunEmphasis: LRE is a measure of the distribution of long run lengths, with a greater value indicative of longer run lengths and more coarse structural textures.

- GLRLM_LongRunHighGrayLevelEmphasis: HGLRE measures the distribution of the higher gray-level values, with a higher value indicating a greater concentration of high gray-level values in the image.
- GLRLM_LongRunLowGrayLevelEmphasis: LRLGLRE measures the joint distribution of long run lengths with lower gray-level values.
- GLRLM_LowGrayLevelRunEmphasis: LGLRE measures the distribution of low gray-level values, with a higher value indicating a greater concentration of low gray-level values in the image.
- GLRLM_RunEntropy: RE measures the uncertainty/randomness in the distribution of run lengths and gray levels. A higher value indicates more heterogeneity in the texture patterns.
- GLRLM_RunLengthNonUniformity: RLN measures the similarity of run lengths throughout the image, with a lower value indicating more homogeneity among run lengths in the image.
- GLRLM_RunLengthNonUniformityNormalized: RLNN measures the similarity of run lengths throughout the image, with a lower value indicating more homogeneity among run lengths in the image. This is the normalized version of the RLN formula.
- GLRLM_RunPercentage: RP measures the coarseness of the texture by taking the ratio of number of runs and number of voxels in the ROI.
- GLRLM_RunVariance: RV is a measure of the variance in runs for the run lengths.
- GLRLM_ShortRunEmphasis: SRE is a measure of the distribution of short run lengths, with a greater value indicative of shorter run lengths and more fine textural textures.
- GLRLM_ShortRunHighGrayLevelEmphasis
- GLRLM_ShortRunLowGrayLevelEmphasis
- GLSZM_ Gray Level Size Zone: quantifies gray level zones in an image. A gray level zone is defined as the number of connected voxels that share the same gray level intensity. A voxel is considered connected if the distance is 1 according to the infinity norm (26-connected region in a 3D, 8-connected region in 2D). In a gray level size zone matrix $P(i,j)$ the (i,j)th element equals the number of zones with gray level i and size j appear in image. Contrary to GLCMand GLRLM, the GLSZM is rotation independent, with only one matrix calculated for all directions in the ROI.
- GLSZM_GrayLevelNonUniformity: GLN measures the similarity of gray-level intensity values in the image, where a lower GLN value correlates with a greater similarity in intensity values
- GLSZM_GrayLevelNonUniformityNormalized: GLNN measures the similarity of gray-level intensity values in the image, where a lower GLNN value correlates with a greater similarity in intensity values. This is the normalized version of the GLN formula.
- GLSZM_GrayLevelVariance: GLV measures the variance in gray level intensities for the zones.
- GLSZM_HighGrayLevelZoneEmphasis: HGLZE measures the distribution of the higher gray-level values, with a higher value indicating a greater proportion of higher gray-level values and size zones in the image.
- GLSZM_LargeAreaEmphasis: LAE is a measure of the distribution of large area size zones, with a greater value indicative of more larger size zones and more coarse textures.
- GLSZM_LargeAreaHighGrayLevelEmphasis: LALGLE measures the proportion in the image of the joint distribution of larger size zones with lower gray-level values.
- GLSZM_LargeAreaLowGrayLevelEmphasis: LAHGLE measures the proportion in the image of the joint distribution of larger size zones with higher gray-level values.
- GLSZM_LowGrayLevelZoneEmphasis
- GLSZM_SizeZoneNonUniformity: SZN measures the variability of size zone volumes in the image, with a lower value indicating more homogeneity in size zone volumes.
- GLSZM_SizeZoneNonUniformityNormalized
- GLSZM_SmallAreaEmphasis: SAE is a measure of the distribution of small size zones, with a greater value indicative of more smaller size zones and more fine textures.

- GLSZM_SmallAreaHighGrayLevelEmphasis: SAHGLE measures the proportion in the image of the joint distribution of smaller size zones with higher gray-level values.
- GLSZM_SmallAreaLowGrayLevelEmphasis: SALGLE measures the proportion in the image of the joint distribution of smaller size zones with lower gray-level values.
- GLSZM_ZoneEntropy: ZE measures the uncertainty/randomness in the distribution of zone sizes and gray levels. A higher value indicates more heterogeneneity in the texture patterns.
- GLSZM_ZonePercentage: ZP measures the coarseness of the texture by taking the ratio of number of zones and number of voxels in the ROI
- GLSZM_ZoneVariance: ZV measures the variance in zone size volumes for the zones.
- NGTDM: A Neighbouring Gray Tone Difference Matrix quantifies the difference between a gray value and the average gray value of its neighbours within distance δ. The sum of absolute differences for gray level i is stored in the matrix.
- NGTDM_Busyness: A measure of the change from a pixel to its neighbour. A high value for busyness indicates a 'busy' image, with rapid changes of intensity between pixels and its neighbourhood.
- NGTDM_Coarseness: Coarseness is a measure of average difference between the center voxel and its neighbourhood and is an indication of the spatial rate of change. A higher value indicates a lower spatial change rate and a locally more uniform texture.
- NGTDM_Complexity: An image is considered complex when there are many primitive components in the image, i.e. the image is non-uniform and there are many rapid changes in gray level intensity
- NGTDM_Contrast: Contrast is a measure of the spatial intensity change, but is also dependent on the overall gray level dynamic range. Contrast is high when both the dynamic range and the spatial change rate are high, i.e. an image with a large range of gray levels, with large changes between voxels and their neighbourhood.
- NGTDM_Strength: Strength is a measure of the primitives in an image. Its value is high when the primitives are easily defined and visible, i.e. an image with slow change in intensity but more large coarse differences in gray level intensities.
